# Pesticides Use Practice by Market Gardeners in Lome (Togo)

**DOI:** 10.1155/2020/8831873

**Published:** 2020-09-22

**Authors:** Aboudoulatif Diallo, Komi Zotchi, Povi Lawson-evi, Batomayena Bakoma, Essotolom Badjabaissi, Eklu-Gadegkeku Kwashie

**Affiliations:** ^1^Department of Toxicology, Faculty of Health Sciences, University of Lome, Lome, Togo; ^2^Department of Animal Physiology, Faculty of Science, University of Lome, Lome, Togo; ^3^Department of Pharmagnosy, Faculty of Health Sciences, University of Lome, Lome, Togo

## Abstract

Pesticides are more and more used in African countries. The aim of this study is to evaluate the risk and the impact of pesticides on gardener's health. It is a transversal descriptive study, which referred to vegetable growers, held in Lomé on the period from May 20 to June 5, 2017. Forty-eight (48) growers participated in the study. Men accounted for 70.8% of the study population against 29.2% of women. The level of education was primary (47.9%) in the majority of cases. Married people or couples represented 77.1% of cases. Most gardeners (72.9%) has no training in the use of pesticides. Pesticides were consistently applied (100%), including insecticides (72.7%), herbicides (9.1%), and fungicides (18.2%). Of 20 pesticides collected, 9 (45%) were approved by our authorities. Only 43.8% of growers were supplied with pesticides from authorized structures of agricultural products. Branches of plants (79.2%) were the spray means of most of the pesticides. Most gardeners (79, 2%) did not use personal protective equipment because of lack of resources for 81.6% of them. Water and environment contamination risk by pesticides was known by only 6.3% of gardeners. Among the adverse effects reported, pruritus was the majority in 85.4% of cases followed by headache (70.8%), cough (68.8%), and muscle pain (64.6%). The training of gardeners, monitoring and control of this informal sector, and the monitoring of unregistered pesticides will help to reduce the risk of poisoning of gardeners and consumers of vegetables as well as environmental pollution.

## 1. Introduction

Market gardening is one of the sectors of agriculture, developed in fruit and vegetable production in urban areas because of the high demand by hotels and households [1, 2]. To increase the yield of the crop and protect it against organisms considered undesirable (clean fresh vegetables with no visible spots or perforations caused by pathogens), pesticides have been heavily used including herbicides, fungicides, and insecticides [3, 4]. The term pesticide refers to plant protection products against all harmful organisms and biocides that are broadly intended to destroy, repel, or render harmless pests. However, according to standards established by the World Health Organization, only safe pesticides should be used in vegetable production [5]. However, the safest pesticides are often more expensive or less effective. It is important to note that phytopharmaceutical products are toxic and their use can only be accepted or encouraged if they are fully controlled in terms of use and the risks to human health and the natural environments that may be affected [6–8]. African countries import less than 10% of the world's pesticides, but they account for half of all accidental poisonings and more than 75% of fatal cases [9].

In Togo, some studies on the use of pesticides in market gardening have been carried out [10, 11]. The particularity of our research is that it assesses the evolution of risky practices and the impact on the health of market gardeners vis-à-vis plant protection products in the Municipality of Lomé by highlighting the pesticides used, the factors of exposure, and the adverse effects of these products.

## 2. Materials and Methods

The study took place in Lomé, on market gardening sites, located on the Ablogame—Avépozo section of the Lomé—Aného road (Togo). This is a descriptive cross-sectional study that covered the period from May 20 to June 5, 2017. A total of 48 market gardeners, all pesticides users, participated in the study. Only market gardeners present during our visit and having freely accepted to participate in our study were included.

A questionnaire structured around the main practices for the use of plant protection products was used for this survey. These include the various products used, the mode of application, the protection of market gardeners during use, and the adverse effects on their health. The sociodemographic characteristics of market gardeners were also evaluated. Interviews were conducted at market gardeners' work place. The questionnaire was administered in the form of an individual interview by the same investigator.

Epi Info 3.1 and Excel 2010 were used for statistical analysis of the data. The variables collected were used to determine frequencies that were compared with each other, using the Khi-2 test, with a significance threshold of 5%.

## 3. Results

### 3.1. Socioprofessional Characteristics

Forty-eight (48) market gardeners participated in the study. Market gardeners were mainly men (70.8%). The average age of market gardeners was 45 years with extremes of 22 and 61 years and a predominance of the age group of 42–52 years. The majority (77.1%) of market gardeners were married or in a relationship. The majority education level was “primary” (46%) followed by “secondary” at 28% (Table 1). The average exercise period was 15.7 years with extremes of 1 to 35 years.

### 3.2. Practices for the Use of Plant Protection Products

All vegetable growers (100%) used pesticides and only 32 market gardeners (66.7%) had given information on the place of supply of their plant protection products. The market was the preferred place for purchasing pesticides (53.1%) compared to 43.8% for approved structures (wholesalers) of agricultural products and 3.1% for the street.

The plant protection products most commonly used by market gardeners were mainly insecticides (72.7%) followed by fungicides (18.2%) and herbicides (9.1%). 37.5 percent of the insecticides were unlicensed compared to 9.9% for herbicides. All fungicides used were unregistered (Table 2).

Based on trade names, 20 pesticides were used by gardeners, of which 9 were registered and 11 were not registered (Tables 3 and 4).

**Figure 1:**
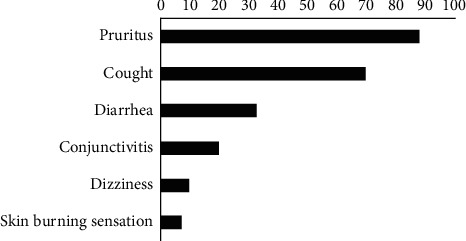
Distribution of market gardeners according to adverse health effects due to pesticides.

Plant branches and leaves were the main route of phytopharmaceutical products application by market gardeners (79.2%). The majority of gardeners (79.2%) did not use personal protective equipment for lack of resources (81.6%), lack of awareness of danger (10.5%), and negligence (7.9%) (Table 5). Only 3 respondents were aware of the risk of pesticide poisoning in water and in the environment. Health problems related to the use of pharmaceuticals encountered by market gardeners have been reported. The majority of adverse effects reported by market gardeners were mainly pruritus (85.4%) followed by headache (70.8%), cough (68.8%), and muscle pain (64.6%) (Figure 1).

## 4. Discussion

The gardeners surveyed were mostly men (70.8%), married or in a relationship (77.1%) and have a low level of education. The low proportion of women (29.2%) was also reported by Mawussi et al. [12] in Togo and by Muliele et al. [13] in Congo. This low proportion of women is due to the fact that this work requires physical effort and women are more involved in the sale of these products.

This low level of education would reflect the lack of knowledge of pesticide application, which obviously leads to improper use or the use of pesticides releasing a lot of residues in harvested vegetables. A low level of education correlated with a lack of training (72.9%) as well as training provided by nonprofessionals promotes the misuse of pesticides [14], since there is a repetition of bad practice. This situation was also observed by Kanda et al. [10] in Lomé (Togo) among market gardeners and in Cotonou (Benin) by Agnandji et al. [15] in 2018. This would be an obvious question since the level of education plays a major role in technological adoption of agricultural innovations. Market gardeners need more training on the management of plant protection products, as our study reported that only 27.1% of respondents had at least a training course in pesticide use. Application parameters such as preharvest period, frequency, and spraying period will not be meticulous and will obviously result in a large amount of residues in the harvested feed. The period left for the degradation of the active ingredients varies from a product to another. But market gardeners do not take it into consideration because they harvest according to the availability of buyers, which will greatly affect the factor that contributes to the existence of residues in the harvested product.

All ages were represented in our study with a predominance of the age group of 42–52 years and extremes ranging from 22 to 61 years. In addition, the average exercise period is 15.7 years. This long exercise period suggests risks of chronic poisoning of market gardeners. Medical monitoring for this informal sector should be considered.

All market gardeners surveyed (100%) used pesticides. The systematic use of synthetic pesticides to optimize vegetable yields has been reported in several other African countries such as Ghana, Togo, Senegal, and Benin [16–19]. The pesticides applied in vegetable crops are numerous and varied. However, in our study, they predominantly belonged to the class of insecticides (72.7%) followed by fungicides (18.2%) and herbicides (9.1%). In contrast, Nkolo market gardeners in the Congo used fungicides, insecticides, and to a lesser extent acaricide insecticides [20]. This difference can be explained by the varieties of vegetables grown and also by the ecology of the environment. 46.3 percent of pesticides in this study were not registered in which 37.5% were the insecticides compared to 9.9% of the herbicides. All the fungicides used were not registered.

The majority of the pesticides used by our respondents were purchased at the market (53.1%) and on the street (3.1%) compared to few in approved structures for plant protection products (43.8%). This explains the use of unregistered pesticides found in our study and this is because of the low cost on the market. These unregistered pesticides could also be at the origin of the negative effects on the health of market gardeners, coupled with the low rate of formation (27.1%). Like medicines, despite the means put in place, the illegal traffic in plant protection products is a real public health problem in Africa where borders are porous. The continuous awareness and training of market gardeners and their support in this culture remain a real alternative to this scourge.

The handling of pesticides is not without risks of intoxication on market gardeners. Some African countries have reported risks of pesticide poisoning on human (with death), animal, and environmental health due to noncompliance with handling conditions [21, 22]. Market gardeners (79.2%) sprayed pesticides with twig leaves, which could also expose them to greater risk of poisoning. The same observation was made in Central Africa where market gardeners used twigs gathered in the form of brooms for spraying [23]. The sprayer is not a tool available to all African gardeners for lack of resources reported by our respondents, which would explain the use of twig leaves for spraying. In addition, nearly 4 out of 5 (79.2%) of the gardeners did not use personal protective equipment for reasons of lack of resources (81.6%), lack of knowledge of the risks (10.5%), and negligence (7.9%). Poor application of pesticides can lead to direct exposure (by eyes, nose, or even skin). De Jaeger et al. [24] in 2012 showed that lack of personal protective equipment can increase the risk of intoxication, which, minor at first, can become serious by bioaccumulation. With regard to protective equipment, the situation was the same in 2013 in Togo [11] where 53% of market gardeners in the Maritime region did not protect themselves.

Several adverse health effects were reported by our respondents, the most important of which were pruritus (85.4%), headache (70.8%), cough (68.8%), and muscle pain (64.6%). This shows the importance of protective equipment when handling and applying pesticides. In addition to the consequences on the health of market gardeners, the accumulation of plant protection products in vegetables and soil can be a source of poisoning. Children of market gardeners, who during weekends and/or during holidays help their parents, are also at great risk of poisoning as well as women in pregnancy who help their husbands.

Several pesticides including aldrin, endosulfan, and dimethoate, are banned in many parts of the world because of their toxicity on the environment and on humans. Unfortunately, they are still used in our countries.

## 5. Conclusion

All market gardeners surveyed used pesticides to increase crop yields. They apply insecticides, herbicides, and fungicides often not approved by the Togolese State. In addition, market gardeners do not have a good knowledge of pesticides and generally do not comply with manufacturers' recommendations, including wearing protective clothing due to their low level of education and lack of training. These results reveal the potential risk of pesticide intoxication faced by market gardeners and vegetable consumers. Awareness of market gardeners must be made so that they will know the risks and they will use personal protective equipment.

## Figures and Tables

**Table 1:** Socioprofessional characteristics of gardeners.

Parameters (*n* = 48)	Characteristics	Number	Number (%)
*Sex*	Man	34	70.8
	Women	14	29.2

*Age*	(22–32)	3	6.2
	(32–42)	14	29.2
	(42–52)	23	47.9
	(52–62)	8	16.7

*Level of education*	Uneducated	10	20
	Primary	23	46
	Secondary	14	28
	Superior	3	6

*Marital status*	Single	11	22.9
	Married/couple	37	77.1

*Agricultural training*	Yes	13	27.1
	No	35	72.9

**Table 2:** Pesticide types, approved or not and used by gardeners.

Type	Registered products (%)	Unregistered products (%)	Total (%)
Insecticides	55 (62.5)	33 (37.5)	88 (72.7)
Herbicides	10 (90.1)	1 (09.9)	11 (9.1)
Fungicides	0 (0)	22 (100)	22 (18.2)
Total	65 (53.7)	56 (46.3)	121 (100)

**Table 3:** List of trade names of pesticides used by growers.

Type	INN (trade name)	Type	Number (%)
Registered products	Tefluthrin (Atac®)	Insecticide/pyrethroid	29 (44.60)
	Emamectin (Emacot®)	Insecticide/pyrethroid	13 (20)
	Cypermethrin (Cypercal®)	Insecticide/pyrethroid	8 (12.31)
	Fenitrothion (Terminator®)	Herbicide/carbamate	8 (12.31)
	Lambdacyhalothrine (Stilambda®)	Insecticide/pyrethroid	2 (3.08)
	Chlorpyrifos-ethyl (Pyriforce®)	Insecticide/organophosphate	2 (3.08)
	Glyphosate (Finish®)	Herbicide/carbamate	1 (1.54)
	Glyphosate (Kalach®)	Insecticide/carbamate	1 (1.54)
	Pendimethalin (Alligator®)	Herbicide/toluidines	1 (1.54)

INN: International Nonproprietary Name.

**Table 4:** List of trade names of pesticides used by growers.

Type	INN (trade name)	Type	Number (%)
Unregistered product	Mancozeb (Mancoezeb®)	Fungicide/carbamate	19 (33.9)
	Carbofuran (Carbofuran®)	Insecticide/carbamate	7 (12.5)
	Lambdacyhalothrine (Sunalothrin®)	Insecticide/pyréthrine	6 (10, 7)
	Chlorpyrifos-Éthyl (Sunpyrifos®)	Insecticide/organophosphoré	6 (10, 7)
	— (Car®)^*∗*^	Insecticide + Fungicide	6 (10, 7)
	Mancozeb (Suncozeb®)	Fungicide/carbamate	3 (5, 3)
	Phenamiphos (Nemacur®^2^)	Insecticide/organophosphoré	2 (3, 6)
	Dimethoate (Dimethoate®)	Insecticide/organophosphoré	2 (3, 6)
	Endosulfan (Endosulfan®)	Insecticide/organochloré	2 (3, 6)
	— (Acaricide®)^*∗*^	Acaricide	2 (3, 6)
	Aldrine (Aldrine®)	Insecticide/organochloré	1 (1, 8)

—: not specified. ^*∗*^The INN (International Nonproprietary Name) is not specified on the packaging.

**Table 5:** Means of implementation and protection of pesticides and knowledge of risks by market gardeners.

Parameters (*n* = number of investigated)	Characteristics	Number (%)
Applying way (*n* = 48)	Branches, leaf	38 (79.2)
	Spray	35 (72.9)
	Spreading	15 (31.3)
	Sprayer	6 (12.5)
	Drip	1 (2.1)
Use of personal protective equipment (*n* = 48)	Yes	10 (20.8)
	No	38 (79.2)
Due to the nonuse of personal protective equipment (*n* = 38)	Lack of means	31 (81.6)
	Ignorance of the danger	4 (10.5)
	Negligence	3 (7.9)
Knowledge of the effects on water and the environment (*n* = 48)	No	30 (62.5)
	Yes	3 (6.3)
	Do not know	15 (31.2)

## Data Availability

The data are not available.
